# Reduced Quality of Life in Patients with Non-Alcoholic Fatty Liver Disease May Be Associated with Depression and Fatigue

**DOI:** 10.3390/healthcare10091699

**Published:** 2022-09-05

**Authors:** Julia A. Golubeva, Anna F. Sheptulina, Adel A. Yafarova, Elvira M. Mamutova, Anton R. Kiselev, Oxana M. Drapkina

**Affiliations:** 1 Laboratory for the Study of Human Gut Microbiota, Department of Fundamental and Applied Aspects of Obesity, National Medical Research Center for Therapy and Preventive Medicine, 101990 Moscow, Russia; 2 Department of Therapy and Preventive Medicine, A.I. Evdokimov Moscow State University of Medicine and Dentistry, 127473 Moscow, Russia; 3 Coordinating Center for Fundamental Research, National Medical Research Center for Therapy and Preventive Medicine, 101990 Moscow, Russia

**Keywords:** non-alcoholic fatty liver disease, quality of life, SF-36, fatigue, depression, lifestyle modifications

## Abstract

Non-alcoholic fatty liver disease (NAFLD) is often thought of as clinically asymptomatic. However, many NAFLD patients complain of fatigue and low mood, which may affect their quality of life (QoL). This may create a barrier to weight loss and hinder the achievement of NAFLD therapy goals. Our study aimed to evaluate the QoL in NAFLD patients vs. healthy volunteers, and to analyze likely influencing factors. From March 2021 through December 2021, we enrolled 140 consecutive adult subjects (100 NAFLD patients and 40 controls). Overall, 95 patients with NAFLD and 37 controls were included in the final analysis. Fatty liver was diagnosed based on ultrasonographic findings. We employed 36-Item Short Form Health Survey (SF-36) to evaluate QoL, Hospital Anxiety and Depression Scale (HADS) to identify anxiety and/or depression, and Fatigue Assessment Scale (FAS) to measure fatigue. NAFLD patients had significantly lower physical component summary scores, as well as significantly higher HADS-D scores, compared with the control group (Mann-Whitney U criterion = 1140.0, *p* = 0.001 and U = 1294.5, *p* = 0.022, respectively). Likewise, fatigue was more common in NAFLD patients (χ2 = 4.008, *p* = 0.045). Impaired QoL was significantly associated with fatigue (FAS score ≥ 22, *p* < 0.001) and depression (HADS-D ≥ 8, *p* < 0.001). In conclusion, NAFLD patients had significantly poorer QoL vs. controls, in particular with respect to the physical component of health. Impaired QoL may be associated with fatigue and depression, and together they may interfere with increased physical activity and lifestyle modifications in patients with NAFLD.

## 1. Introduction

At present, non-alcoholic fatty liver disease (NAFLD) has become the most common cause of chronic liver disease, and its global prevalence among adults worldwide is approximately 25% [1]. The prevalence of NAFLD is increasing rapidly, posing a major public health concern [2].

Currently, the most effective and recognized treatment for NAFLD is focused on lifestyle modifications aimed at changing body composition through physical activity, control of a sedentary lifestyle, and diet. Nevertheless, K.E. Stewart, et al. (2015) in their study, aimed to evaluate the readiness to change weight-related behaviors in patients with NAFLD, found that only 10% of participants (6 out of 58) were either preparing to or actively making lifestyle changes six months after consultation with a physician, even though all participants received nutritional education and guidance [3]. Authors also showed that depression, low conscientiousness, and high neuroticism were associated with higher weight at a six-month follow-up. These data are consistent with the results of another study, showing that the coexistence of major depressive disorder and NAFLD is associated with worse treatment outcomes in patients with NAFLD [4].

Depression, as well as increased fatigue, and anxiety are thought to be more prevalent in patients with NAFLD, compared to the general population [5,6,7]. Indeed, according to the results of the study by C. Labenz, et al. (2020), including 19,871 patients with NAFLD and 19,871 matched controls, followed-up for 10 years, NAFLD constitutes an independent risk factor for emerging depression and anxiety even after controlling for confounding comorbidities (diabetes mellitus, cardiovascular diseases, asthma/chronic obstructive pulmonary disease, and cancer) [8]. Moreover, growing evidence suggests that, along with anxiety and depression, several other central nervous system (CNS) disorders may be significantly associated with NAFLD, for example, cognitive impairment, hippocampal-dependent memory impairment [5], as well as alteration of rapidity and precision of executive functions and a higher degree of behavioral disturbances [9]. Additionally, R. Moretti, et al. (2022) demonstrated a strong correlation between NAFLD and subcortical vascular dementia, which may be explained at least in part by the association of NAFLD with widely accepted risk factors of this CNS disorder, such as folate, vitamin B12, and vitamin D deficiency, as well as increased levels of homocysteine [10].

The other important issue limiting motivation and readiness for behavioral changes in patients with NAFLD is the impaired quality of life (QoL), apparent even in patients with early stages of the disease. As to the factors, that can be linked to poor QoL in NAFLD, a recently published systematic review showed that the presence of liver cirrhosis and fatigue may be the major contributors [11]. Further, according to the data obtained by N. Samala, et al. (2020), the quality of life in NAFLD patients may depend on body composition. It was demonstrated that a greater amount of lean body mass was associated with better QoL [12].

Most studies on the association of NAFLD with depression and/or anxiety were aimed at investigation of pathophysiological bases of NAFLD and mood disturbances comorbidity, at determining risk factors of depression and/or anxiety in NAFLD patients, as well as at evaluation of their possible impact on the natural history of NAFLD and treatment outcomes. However, to the best of our knowledge, currently there are no published studies, assessing the contribution of mood disturbances to impaired QoL in patients with NAFLD.

Our study aimed to evaluate the QoL in NAFLD patients vs. control subjects via assessing the prevalence of fatigue, depression, and anxiety as possible causative factors of low QoL, and their relationship with the latter.

## 2. Materials and Methods

### 2.1. Study Participants

This was a prospective cross-sectional single-center study. We calculated that the total sample size in this study should be not less than 133 at an alpha of 0.05 and a power of 0.80 [13]. During the sample size calculation, we took into consideration unequal sample sizes (with an approximate allocation ratio of 3:1) due to the high prevalence of NAFLD [14]. Therefore, during the period from March 2021 through December 2021, we enrolled 140 consecutive adult subjects, aged 18–75 years old, who seek medical care from a physician or gastroenterologist at the Clinical and Diagnostic Division of the National Medical Research Centre for Therapy and Preventive Medicine, and for whom the inclusion criteria for this study were met.

For patients with NAFLD inclusion criteria were as follows: age from 18 to 75 years old, Fatty Liver Index (FLI) of 60 or greater, as well as ultrasound findings consistent with fatty liver (see below), and a signed informed consent form. For the control group, the following inclusion criteria applied: age from 18 to 75 years old, the absence of fatty liver disease sensu FLI values < 60 and ultrasound data, and a signed informed consent form.

Subjects were excluded from the study if they had a history of the following diseases (or any of the following diseases/conditions were identified during the examination): comorbid liver diseases (including viral hepatitis B and C, Wilson disease, autoimmune hepatitis, primary sclerosing cholangitis, primary biliary cholangitis, haemochromatosis), hepatocellular carcinoma, and malignancies over the last five years, psychiatric disorders, morbid obesity, type 1 or 2 diabetes mellitus, chronic obstructive pulmonary disease, bronchial asthma, acute infectious diseases, exacerbation of chronic non-communicable diseases (within four weeks prior to participation in the study), chronic kidney disease of the stage IIIB or more severe (GFR < 30 mL/min/1.73 m^2^). In addition, we did not include subjects who were vegan or vegetarian and subjects with a history of alcohol abuse or hepatotoxic medication use. Also, pregnant and breastfeeding women were not allowed to participate. Similar exclusion criteria were applied to the control group.

Interviews, collection of anthropometric data (weight, height, body mass index), laboratory tests, ultrasound examination of the abdominal cavity, and filling in questionnaires were carried out on the same visit. All questionnaires belonged to the time period of the previous month.

### 2.2. Assessments

#### 2.2.1. Assessment of Fatty Liver Disease

Fatty liver disease was diagnosed on the basis of characteristic ultrasonographic (USG) findings consistent with parenchymal brightness, bright liver pattern (liver-kidney contrast), posterior attenuation, and impaired visualization of the intrahepatic vessels.

We also calculated Fatty Liver Index (FLI) using the following expression [15]:

FLI = (e^0.953 × ln(triglyceride) + 0.139 × BMI + 0.718 × ln(GGT) + 0.053 × waist circumference − 15.745^)/(1 + e^0.953 × ln(triglyceride) + 0.139 × BMI + 0.718 × ln(GGT) + 0.053 × waist circumference − 15.745^) × 100,

where BMI is body mass index and GGT is gamma-glutamyl transferase.

We used the cutoff value of FLI ≥ 60 to rule in fatty liver disease with a positive likelihood ratio of 4.3 [15]. Solely patients with USG results indicative of fatty liver disease and FLI ≥ 60 were included in the study.

In order to exclude advanced liver fibrosis, fibrosis-4 (FIB-4) score was calculated using the following expression [16]:

FIB-4: age (years) × AST [U/L]/(platelets [10^9^/L] × (ALT [U/L])^1/2^),

where AST is aspartate aminotransferase and ALT is alanine aminotransferase.

The value of FIB-4 < 1.3 was used to exclude the presence of advanced liver fibrosis with a negative predictive value of >90% [16].

#### 2.2.2. Assessment of Fatigue

To evaluate fatigue, we used the Fatigue Assessment Scale (FAS), as it measures all aspects of fatigue, representing both physical and mental symptoms, independently from depression and neuroticism. Because of its psychometric properties, brevity, and ease of administration, the FAS is a valuable tool for assessing fatigue, and can be used in clinical research as a validated fatigue assessment measure [17]. It was shown that FAS had good responsiveness in chronic hepatitis C [18], and is suggested to be used for fatigue measurement in patients with chronic liver diseases [19]. This questionnaire consists of 10 statements evaluating mental and physical fatigue. The results could range from 10 to 50 points. An overall score of 22 points or greater was indicative of clinically significant fatigue [20].

#### 2.2.3. Assessment of Anxiety and Depression

To measure anxiety and depression, we used the Hospital Anxiety and Depression Scale (HADS), a questionnaire validated for epidemiological and clinical studies. This scale was designed specifically for the assessment of anxiety and depression in non-psychiatric medical outpatients and community populations [21], and has been demonstrated to be reliable when applied to patients with chronic liver diseases [22]. The overall cumulative score was calculated separately for anxiety (HADS-A) and depression (HADS-D). Individuals were coded as having depression/anxiety if they had a HADS-D/HADS-A score of ≥8 [23].

#### 2.2.4. Quality of Life Assessment

Quality of life was measured via the 36-Item Short Form Health Survey (SF-36). This is a standardized tool for assessing the quality of life, validated in the general population as well as in people with multiple chronic medical conditions yielding good psychometric properties. The SF-36 was chosen to assess QoL for this study for the following reasons. First, it is used widely in the literature in different disease states with well-established interpretive methodology and normative data. Second, as we aimed to compare the QoL in NAFLD patients and in healthy volunteers, it was crucial to utilize an instrument that was validated in both the general population and in subjects with multiple chronic medical illnesses. The SF-36 has already shown good performance in the NAFLD population [24]. This questionnaire consists of 36 items grouped into eight categories (subscales): physical functioning (PF), role limitations due to physical health problems (RP), bodily pain (BP), general health perceptions (GH), vitality (energy/fatigue, VT), social functioning (SF), role limitations due to personal or emotional problems (RE), and mental health (emotional wellbeing, MH). The subscales are grouped into two composite components: physical component summary (physical functioning, role physical, bodily pain, general health) and mental component summary (vitality, social functioning, role emotional and mental health), PCS, and MCS, correspondingly. The scores are converted to a scale from 0 to 100, allowing the scales to be measured numerically, with higher scores implying better health [25].

### 2.3. Statistical Analysis

Data were analyzed with IBM SPSS Statistics Version 28.0 (IBM Corp., Armonk, NY, USA). We calculated that the sample size in this study should be not less than 133 at an alpha of 0.05 and a power of 0.80 [13]. During the sample size calculation, we took into consideration unequal sample sizes (with an approximate allocation ratio of 3:1) due to the high prevalence of NAFLD [14]. The normality of data distribution was investigated using the Kolmogorov–Smirnov test. Because of non-normally distributed data, nonparametric tests were applied to our data. Descriptive statistics included medians and interquartile ranges (IQR) for continuous variables, along with frequencies and percentages for categorical variables. To estimate intergroup differences, we employed the Mann-Whitney U test and Spearman’s rank correlation coefficient for continuous parameters. For categorial parameters, Pearson’s chi-squared test (χ2) was used. A logistic regression analysis was performed to identify independent predictors associated with QoL deterioration. For all analyses, two-sided statistical significance was determined as *p* < 0.05.

## 3. Results

We recruited 140 consecutive subjects. Non-alcoholic fatty liver disease was diagnosed in 100 subjects, and 40 subjects were allocated to the control group. The fact that the NAFLD group included nearly a three-times greater number of subjects compared to the control group complied with the prevalence of NAFLD in our country [26]. Seven patients were excluded from the NAFLD group for the following reasons: three patients—due to hyperglycemia and abnormal glucose tolerance test’s results, one patient—due to the positive result of the test for anti-HCV antibodies, and one patient did not come to the center for a scheduled visit, and we were unable to reach this patient by phone. As to the control group, three subjects were excluded from the final analysis: one patient —due to the positive result of the test for HBsAg, one patient —due to the positive result of the test for anti-HCV antibody, and one patient—due to the refusal to fill in the questionnaires. Overall, 95 patients with NAFLD and 37 controls were included in the final analysis.

Anthropometric and biochemical parameters for both groups are presented in the comparative form in Table 1. In NAFLD patients, results of blood biochemistry tests were significantly different from those in controls, and complied with the natural history and specific biochemical profile of this liver disease. Moreover, patients with NAFLD in our study were older, than the control subjects, and this corresponds to the literature data on the increasing prevalence of NAFLD with age [27].

**Figure 1:**
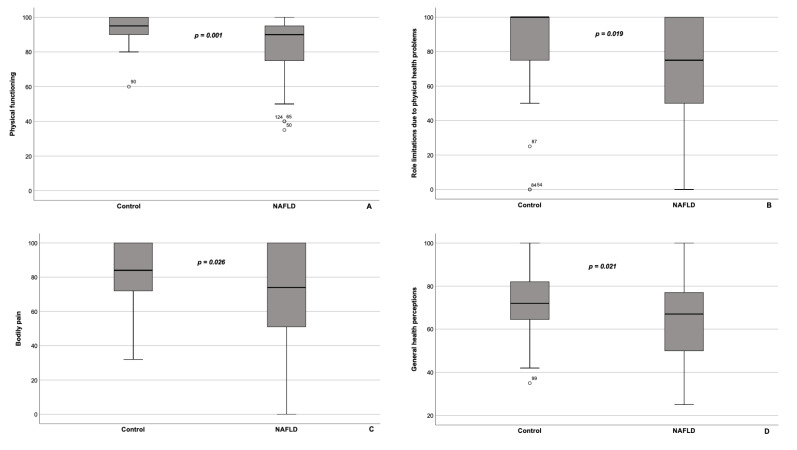

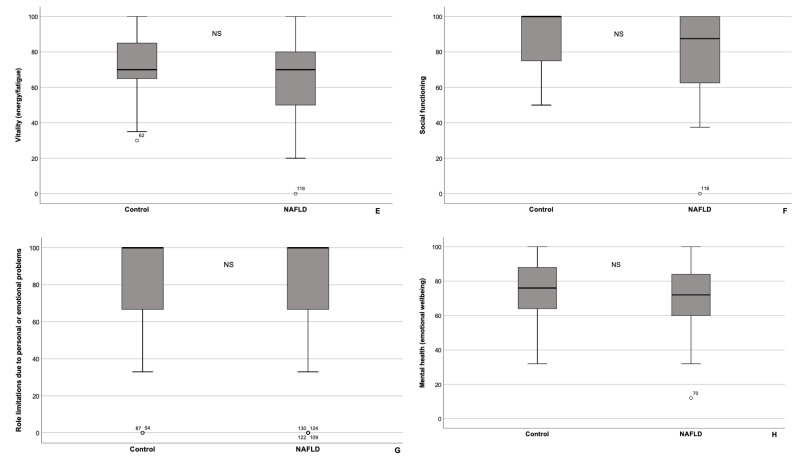
Box Plot of the SF-36 questionnaire subscale values for patients with NAFLD (*n* = 95) and controls (*n* = 37). Physical functioning (**A**), role limitations due to physical health problems (**B**), bodily pain (**C**), and general health perceptions (**D**) comprise the physical component summary. Vitality (**E**), social functioning (**F**), role limitations due to personal or emotional problems (**G**), and mental health (**H**) comprise the mental component summary. Patients with NAFLD had significantly lower scores on all SF-36 categories, comprising the physical component summary score (i.e., physical functioning (**A**), role limitations due to physical health problems (**B**), bodily pain (**C**), and general health perceptions (**D**)). On the contrary, scores on the subscales, included in the mental component summary (i.e., vitality (**E**), social functioning (**F**), role limitations due to personal or emotional problems (**G**), and mental health (**H**)), tended to be higher in patients with NAFLD, though the differences did not reach statistically significant level. The line through the middle of each box represents the median. The length of the box thus represents the interquartile range. The error bars show the minimum and maximum values of each subscale. Outliers are depicted as circles. All comparisons are performed using Mann-Whitney U test. NS—non-significant.

SF-36 survey yielded the following results: NAFLD patients had significantly lower PCS scores (48.46 [37.2–55.0] vs. 54.12 [47.42–58.87], *p* = 0.001), and scores on all subscales, constituent of the PCS, namely PF, RP, BP, and GH, compared with the control group (Figure 1A–D). On the contrary, the MCS scores did not differ substantially between the groups, but were higher in patients with NAFLD than in controls (51.20 [43.53–56.22] vs. 45.81 [40.56–54.28], respectively, *p* = 0.098). As to MCS subscales, there were no significant differences between VT, SF, RE, and MH scores between NAFLD patients and controls (Figure 1E–H). It is worth noting that in the NAFLD group scores on PF and RP subscales, which are parts of PCS, correlated significantly with the BMI (Spearman’s ρ = −0.280, *p* = 0.006 and ρ = −0.255, *p* = 0.013, respectively), whereas in the control group there were no relevant correlations between BMI and SF-36 categories’ values.

A comparison of FAS scores also revealed that NAFLD patients were more likely to have clinically significant fatigue than control subjects (46.7% vs. 28.9%, respectively, χ2 = 4.008, *p* = 0.045).

**Figure 2:**
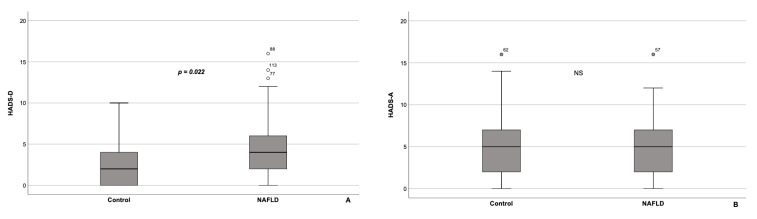
Box Plot of the HADS-D (**A**) and HADS-A (**B**) score values for patients with NAFLD (*n* = 95) and controls (*n* = 37). Patients with NAFLD had significantly higher HADS-D scores, compared with the controls (**A**), whereas HADS-A scores did not differ substantially between the groups (**B**). The line through the middle of each box represents the median. The length of the box thus represents the interquartile range. The error bars show the minimum and maximum values of HADS-D and HADS-A scores. Outliers are depicted as circles. HADS-A—Hospital Anxiety and Depression Scale, subscale for anxiety; HADS-D—Hospital Anxiety and Depression Scale, subscale for depression. All comparisons are performed using Mann-Whitney U test. NS—non-significant.

According to HADS scores, patients with NAFLD had significantly higher HADS-D scores, compared with the controls (Figure 2A). At the same time, the prevalence of anxiety did not differ significantly between NAFLD patients and control subjects (Figure 2B). In addition, we revealed a positive correlation between the HADS-D scores and the FIB-4 index values (ρ = 0.206, *p* = 0.048). However, the comparison of HADS-D scores between the subgroups of NAFLD patients with significant fibrosis (F ≥ 3 on METAVIR scale; FIB-4 ≥ 1.3, *n* = 18) and without it (FIB-4 < 1.3, *n* = 74) yielded no statistically significant differences. Moreover, there were significant negative correlations between FIB-4 values and PF, as well as BP subscales’ scores (parts of PCS) in the NAFLD group (ρ = −0.343, *p* < 0.001 and ρ = −0.229, *p* = 0.027, respectively)**.** In the control group, we discovered 6 subjects with FIB-4 > 1.3.

When analyzing factors influencing the QoL of NAFLD patients, we established that clinically significant fatigue and depression were associated with lower QoL scores on all subscales. Quite similar results were obtained for the control group (Table 2). Though, Spearman’s rank correlation coefficients were higher for the association between PF and FAS scores, VT and FAS scores, SF and FAS scores, RE and HADS-D scores, as well as MH and FAS scores in controls compared to patients with NAFLD, the value of the respective correlation coefficients in controls and in patients with NAFLD belonged to the same strength category. Indeed, a moderate negative association was identified between PF and FAS scores, SF and FAS scores, RE and HADS-D scores, as well as MH and FAS scores, and a strong negative association was detected between VT and FAS scores in both groups.

As for logistic regression analysis, we did not find independent associations between fatigue and certain variables (BMI, depression, anxiety, and SF-36 subscales), which are the components of MCS, in both groups—probably, due to the limited sample size. However, based on stepwise regression analysis, clinically apparent fatigue was associated with RE and VT subscales, as well as with the PCS in patients with NAFLD (R^2^ = 0.557, *p* < 0.001). Additionally, when we used the presence of depression as a dependent variable, we identified a significant association with the RP subscale, a component of PCS (R^2^ = 0.262, *p* < 0.001).

## 4. Discussion

Increasing evidence suggests deterioration of QoL in patients with chronic liver diseases, regardless of their etiology, and also in patients with metabolic syndrome [5,25]. Although data regarding the impact of NAFLD on health-related QoL are limited, an increasing number of publications indicates the tentative impact of NAFLD on this parameter [24].

In this observational case-control study involving 132 participants with complete data (95 patients with NAFLD and 37 healthy volunteers), patients with NAFLD had significantly poorer QoL vs. the control group, in particular with respect to the physical component of health. Similarly, the study performed by A. Afendy, et al. (2009) demonstrated that patients with NAFLD exhibited significantly poorer QoL than patients with other liver diseases included in that study (alcoholic fatty liver disease, autoimmune hepatitis, viral hepatitis B and C, cholestatic liver disease). Authors attributed such results to the fact that nearly all patients with NAFLD were overweight or obese. Besides, NAFLD is a part of the metabolic syndrome; and other manifestations of that syndrome, in particular arterial hypertension, diabetes mellitus, and hyperlipidemia, may also contribute to QoL impairment [28]. Investigating the individual impact of each of these comorbidities is problematic due to the strong association between them. Our study did not include patients with NAFLD and diabetes mellitus, and we were able to demonstrate that patients with NAFLD without diabetes mellitus had significantly lower QoL than healthy individuals as well. The presence of arterial hypertension, its degree, and its stage were not specifically explored in our study.

Further, we assessed the impact of BMI on the QoL in patients with NAFLD and controls. The BMI of NAFLD patients was substantially higher than that in the control group, and there were significant negative correlations between BMI and scores on PF, and RF subscales, both of which were parts of PCS, in the NAFLD group. On the contrary, we did not find any relevant correlations between BMI and SF-36 categories’ values in the control group. Our data are consistent with the results of the National multicenter cross-sectional survey in China, indicating that QoL in patients with NAFLD deteriorated with the increase in BMI, the latter being an independent risk factor. In this study, QoL was measured with Chronic Liver Disease Questionnaire (CLDQ) score. However, even though NAFLD patients with normal BMI had higher CLDQ scores vs. overweight or obese NAFLD patients, their QoL remained poor as well [29].

Currently, increasing evidence suggests a strong association between NAFLD/NASH and psychological disorders, such as depression [30,31]. We found out that patients with NAFLD had significantly higher scores on HADS-D, compared with the controls (4 [2–6] vs. 2 [0–4], respectively). Our data are consistent with the results of a recent meta-analysis of observational studies, which showed that patients with NAFLD had a significantly increased risk of depression (pooled odds ratio [OR] = 1.13, 95% CI: 1.03–1.24, *p* = 0.007) [6]. Apparently, the high prevalence of depressive disorder among patients with NAFLD has a negative impact on the QoL: we revealed a significant negative correlation between depression and QoL in patients with NAFLD. The importance of our results here is that these data apply to relatively young patients with NAFLD (median age was 50 years, interquartile range: 43–58 years).

Additionally, we analyzed the effect of liver fibrosis on depression severity and discovered a statistically significant positive correlation between HADS-D, FIB-4, and FLI scores. In a large study including 112,797 NAFLD patients from Seoul, after adjusting for BMI, physical activity, and insulin resistance index (HOMA-IR), the authors detected a similar relationship [32]. However, when participants were stratified by FIB-4 score and assessed for the association of FIB-4 with depression, no statistically significant association was found, which was also consistent with the results of our study. These data could be explained by the low percentage of individuals with significant liver fibrosis in the study group. In addition, we discovered 6 subjects with FIB-4 > 1.3 in the control group. They had normal levels of ALT, AST, and platelet count, and the increase in FIB-4 was apparently related to their age. This finding emphasizes the bias of this method and the need to use it solely for screening purposes.

According to our results, the prevalence of anxiety did not differ significantly between patients with NAFLD and controls. On the contrary, C. Labenz et al. (2020) showed that the hazard ratio for the incidence of anxiety in patients with NAFLD was 1.23 (*p* <0.001), though, in the subgroup analysis, this association remained significant only in women (*p* <0.001), but not in men (*p* <0.067) [8]. Similar results were demonstrated by J.M. Choi et al. (2021). In their study severe NAFLD (determined as a marked increase in bright echoes with poor or no visualization of intrahepatic vessel borders, the diaphragm, and the posterior right lobe of the liver on the abdominal ultrasound) significantly correlated with state anxiety and trait anxiety solely in women (adjusted OR 1.84 and 95% CI 1.01–3.37, *p* = 0.047 and adjusted OR 2.45 and 95% CI 1.08–4.85, *p* = 0.018, respectively) [33]. Such a discrepancy in the results of our study and the above-mentioned studies may be explained at least in part by the small sample size in our study, as the effect size of anxiety, found in the above-mentioned studies was not very large.

Another factor associated with lower QoL, according to our results, is severe fatigue. The authors of a systematic review focusing on the QoL in NAFLD patients also concluded that since fatigue was a common symptom among patients with NAFLD, it could account for poor physical health outcomes [11]. Asthenic syndrome and one of its manifestations, fatigue, is the most common complaint of patients with NAFLD. Our data confirmed that fatigue was nearly twice as common in patients with NAFLD vs. those without it. The combination of factors, such as metabolic disorders and chronic inflammation, occurring in hepatic steatosis, creates pathways for the development of various types of fatigue. The most common types of fatigue mentioned in the literature are central and peripheral fatigue. Central fatigue is characterized by a lack of self-motivation, while peripheral fatigue typically manifests itself in neuromuscular dysfunction and muscle weakness [34]. Some studies suggested that both types of fatigue were present in patients with chronic liver disease [35,36]. Therefore, fatigue can manifest itself as a lack of intention and inability to exercise, which may be the cornerstone of treatment for overweight/obese patients with NAFLD.

In our study, we showed that FAS scores correlated negatively with scores on all the SF-36 subscales. Unsurprisingly, similar negative correlations were observed in the control group (though, not for all SF-36 categories), suggesting uniform unfavorable impact of depression and fatigue on the quality of life. However, when we compared the QoL of NAFLD patients and controls, we confirmed the significant difference in the physical component summary values between the groups, indicating substantially lower QoL in terms of the PCS in NAFLD patients compared to controls. Moreover, our data suggest the negative impact of depression and fatigue on the QoL in NAFLD patients. This justifies the relevancy of depression and fatigue screening and assessment in patients with NAFLD in order to enhance the treatment strategies, patient adherence to recommendations on lifestyle modification, and improve treatment outcomes. Our data may be of greater importance, taking into account the insufficient or low readiness of NAFLD patients to change behavior in order to achieve the treatment goals. Thus, we suggest looking from a different perspective at the problem of reduced motivation for lifestyle changes, weight loss through exercise, and dietary modifications in patients with NAFLD.

Another important issue is that depressed patients have a significantly increased risk of developing NAFLD, compared with patients without depression (pooled OR = 1.46, 95% CI: 1.15–1.85, *p* = 0.002) [37]. Taken together, these data suggest a potential bidirectional relationship between NAFLD and depression, the pathogenesis of which needs to be studied in detail.

According to the results of the logistic regression analysis, clinically apparent fatigue was associated with scores on RE and VT subscales, which are components of MCS, as well as with PCS scores in patients with NAFLD in our study. These findings are in line with the data of a systematic review performed by K. Assimakopoulos, et al. (2018), showing that fatigue, as well as cirrhosis, are the main contributors to impaired QoL in patients with NAFLD [11]. Additionally, when we used the presence of depression as a dependent variable, we identified a significant association with the RP subscale, a component of PCS. To the best of our knowledge this correlation, was not previously described.

Besides depression and anxiety, several other CNS disorders are thought to be associated with NAFLD, particularly cognitive impairment, hippocampal-dependent memory impairment [5], alteration of rapidity and precision of executive functions, a higher degree of behavioral disturbances [9], and subcortical vascular dementia [10]. In a recently published cross-sectional study, comprising 285 patients with subcortical vascular dementia, it was demonstrated that there is a positive correlation between NAFLD se-verity and higher apathy, anxiety, behavioral worsening, and decreased quality of life, as well as a negative correlation with the presence of executive dysfunctions.

Due to the possible negative impact of depression, fatigue, and the above-mentioned CNS disorders on the treatment outcomes of patients with NAFLD, further studies are needed to elucidate the possible underlining mechanisms of these associations, and to identify effective pharmacological targets.

## 5. Conclusions

In conclusion, we should point out that patients with NAFLD suffer from significant impairment of their QoL, related to physical health. The presence of a depressive disorder and fatigue significantly contributes to the deterioration of the quality of life in patients with NAFLD, which could become an obstacle to increasing physical activity and lifestyle modifications.

Evaluation and timely treatment of mental health, as well as fatigue therapy in NAFLD patients, could constitute a promising direction in terms of increasing patient adherence to doctor’s recommendations, and improving the efficacy of therapy, long-term prognosis, and QoL in this group of patients.

## 6. Limitations

This study has some limitations. First of all, it was a single-center study with a relatively small sample size, though the participants comprised a representative sample of NAFLD patients in Russia. Second, cases and controls were not matched in the age category. Third, our patients were not examined by a psychiatrist, and a clinical psychologist to confirm the depressive disorder. Clinical interviews have disadvantages when widely used due to significant time expenditure and cost; hence, we aimed solely to estimate the prevalence of psychological disorders. Additionally, we admit the possibility that in parallel with the adverse impact of depressive disorder and fatigue, QoL in patients with NAFLD may be negatively influenced by other factors, including but not limited to high BMI, metabolic syndrome, concomitant chronic non-communicable diseases, etc. In support of this conjecture, in a recently published study by N. Samala, et al. (2020) authors have shown that on multivariable analysis, SF-36 physical functioning scores were negatively associated with type 2 diabetes, body mass index, and liver stiffness measurement and positively associated with lean body mass and level of alanine aminotransferase [12]. In our study, there were significant negative correlations between BMI, PF, and RF subscales’ scores (parts of PCS), as well as between FIB-4 values, PF, and BP subscales’ scores (parts of PCS) in the NAFLD group. Although, according to the logistic regression analysis only FAS and HADS-D scores were significantly associated with the components of PCS. Our study did not include patients with NAFLD and diabetes mellitus, and we were able to demonstrate that patients with NAFLD without diabetes mellitus had significantly lower QoL than control subjects as well.

Despite these limitations, our study shows that patients with NAFLD have poorer QoL, related to physical health, compared to controls, and that depressive disorder and fatigue significantly contributes to the deterioration of the quality of life in these patients.

## Figures and Tables

**Table 1:** Anthropometric and biochemical parameters of patients with NAFLD and controls.

Parameter	NAFLD	Controls	*p*
Number	95	37	
Gender: female, *n* (%)	59 (62.1)	29 (78.4)	0.075 ^a^
Age (years)	50 (43–58)	45.5 (36–51)	0.008 ^b^
BMI, (kg/m^2^)	30.92 (28.34–34.62)	23.29 (21.04–26.36)	<0.0001 ^b^
Normal weight (BMI < 25 kg/m^2^), *n* (%)	4 (4.2)	24 (64.9)	<0.0001 ^a^
Overweight (BMI ≥ 25 and <30 kg/m^2^), *n* (%)	34 (35.8)	12 (32.4)	0.635 ^a^
Obese (BMI ≥ 30 kg/m^2^), *n* (%)	57 (60.0)	1 (2.7)	<0.0001 ^a^
Platelet count (×10^9^/L)	254 (207–289)	239 (208.5–255.25)	0.087 ^b^
ALT (IU/L)	23 (17–35)	13.5 (10.0–24.0)	<0.0001 ^b^
AST (IU/L)	21 (18–26)	18 (15.75–23.25)	0.012 ^b^
GGT (IU/L)	30 (21–50)	17 (13–30)	<0.0001 ^b^
Glucose (mmol/L)	5.7 (5.2–6.3)	5.3 (5.07–5.72)	0.005 ^b^
Triglycerides (mg/dL)	1.47 (1.08–2.08)	0.88 (0.67–1.28)	<0.0001 ^b^
Fibrinogen (g/L)	3.8 (3.4–4.2)	3.45 (3.2–3.97)	0.014 ^b^
CRP (mg/L) (mg/L)	1.7 (0.9–3.4)	1.02 (0.38–1.84)	<0.0001 ^b^
Uric acid (mg/dL)	5.9 (4.9–7)	4.6 (3.9–5.9)	<0.0001 ^b^
Insulin (µIU/mL)	11.7 (8.47–14.52)	5.7 (4.8–7.7)	<0.0001 ^b^
HOMA-IR > 2.7, *n* (%)	53 (55.7%)	2 (5.4%)	<0.0001 ^a^
TyG	8.84 (8.49–9.18)	8.25 (7.88–8.65)	<0.0001 ^b^
FLI > 60, *n* (%)	65 (69.1%)	0	<0.0001 ^a^
VAI	1.88 (1.17–2.88)	0.91 (0.58–1.19)	<0.0001 ^b^

Presented values denote frequency (%) or median (interquartile range). BMI: body mass index, ALT: alanine aminotransferase, AST: aspartate aminotransferase, GGT: gamma-glutamyl transferase, CRP: C-reactive protein, HOMA-IR: homeostasis model assessment of insulin resistance, TyG: triglyceride glucose index, FLI: fatty liver index, VAI: visceral adiposity index. ^a^ Pearson’s chi-squared test (χ2); ^b^ Mann-Whitney U test.

**Table 2:** Spearman’s rank correlation coefficients between HADS-D scores, FAS scores, and SF-36 subscales’ scores in patients with NAFLD and controls.

**NAFLD Group (*n* = 95)**								
	**PHYSICAL COMPONENT** **SUMMARY**				**MENTAL COMPONENT** **SUMMARY**			
**SF-36** **SUBSCALES**	**PF**	**RP**	**BP**	**GH**	**VT**	**SF**	**RE**	**MH**
HADS-D	−0.526 *	−0.518 *	−0.307 *	−0.498 *	−0.620 *	−0.571 *	−0.453 *	−0.605 *
FAS	−0.564 *	−0.463 *	−0.314 *	−0.497 *	−0.745 *	−0.477 *	−0.528 *	−0.578 *
**Control group (** ***n* = 37)**								
	**PHYSICAL COMPONENT** **SUMMARY**				**MENTAL COMPONENT** **SUMMARY**			
**SF-36** **SUBSCALES**	**PF**	**RP**	**BP**	**GH**	**VT**	**SF**	**RE**	**MH**
HADS-D	−0.460 **	−0.268	−0.095	−0.568 **	−0.622	−0.495 **	−0.484 **	−0.570 **
FAS	−0.641 **	−0.371 *	−0.189	−0.397 *	−0.800 **	−0.513 **	−0.487 **	−0.668 **

* *p* < 0.05; ** *p* < 0.01; *p*-value denotes the significance of correlation. PF—physical functioning, RP—role limitations due to physical function, BP—bodily pain, GH—general health, VT—vitality, SF—social functioning, RE—role limitations due to emotional health, MH—mental health. The physical component summary, characterizing the physical component of health, includes the following SF-36 subscales: PF, RP, BP, and GH. The mental component summary, characterizing the mental component of health, includes the following SF-36 subscales: VT, SF, RE, and MH.

## Data Availability

The data presented in this study are available on request from the corresponding author. The data are not publicly available due to privacy restrictions.
